# *In Operando* Characterization of Nanocellulose Based Water Treatment Materials Using Atomic Force Microscopy and Synchrotron Scattering

**DOI:** 10.1021/accountsmr.5c00150

**Published:** 2025-11-25

**Authors:** Houssine Khalili, Senuri Kumarage, Aji P. Mathew

**Affiliations:** † Department of Chemistry, 7675 Stockholm University, Svante Arrhenius väg 16 C, Stockholm SE-106 91, Sweden; ‡ Stockholm University Center for Circular and Sustainable Systems (SUCCeSS), Stockholm University, Stockholm SE-106 91, Sweden

## Abstract

Nanocellulose in anionic and cationic form can be extracted from biomass using a top-down approach, and the surface chemistry can be tuned to have selective interactions toward water pollutants under aqueous conditions. The versatility of the surface functionalization potential of nanocellulose and its processability into membranes, hydrogel beads, 3D printed filters, electrospun webs, etc., have resulted in promising performance in water treatment. Nanocellulose interactions with pollutants and adsorption can involve multiple mechanisms such as electrostatic interactions, complexation, hydrophobic interactions, hydrogen bonding, precipitation, or nucleation and growth depending on time scales. This is, however, not fully understood, predominantly due to challenges related to characterization under aqueous conditions. In this context, we explored liquid phase atomic force microscopy (AFM), colloidal probe force spectroscopy, and *in situ* synchrotron scattering methods as advanced characterization tools to extract reliable information on interactions of nanocellulose with metal ions, dyes, pesticides, pharmaceuticals, humic acid, nitrates, PFAS, microplastics, proteins, bacteria, etc., under aqueous conditions. AFM provides information on structure and nanomechanics data on length scales of 1 nm to microns as well as molecular level interactions, whereas scattering methods can detect structures in the range of 1 Å–100 nm. This Account summarizes the research using these techniques under *in operando* conditions to understand reactions and interactions under aqueous conditions for nanocellulose based systems in the context of water treatment. The use of these techniques to understand the adsorption process, membrane structure, and interactions in wet environments, as well as the synthesis of water treatment materials in aqueous media, is included in this Account. In addition to our work, other relevant reports in the literature are also summarized to demonstrate the possibilities and challenges in this approach. Literature review showed only 6 studies on using AFM/force spectroscopy (4 from our group) and only 3 studies (from our group) on scattering methods on nanocellulose in water treatment, which indicates the challenges and limitations of this approach and also the need for expanding this field.

Our works in this field have demonstrated that the advanced characterization methodologies discussed here, viz., atomic force microscopy and X-ray scattering, have significant potential to provide information on nano, molecular, and atomic scales. It is worth mentioning that in order to compensate for the interference with water, which can reduce the accuracy of the data, careful tailoring of experimental design and method development is needed. We also infer that these methodologies and tools, developed to evaluate how the nanocellulose surface interacts/reacts with other hybrid components, biomolecules, and pollutants, can be extended to understand materials and devices (e.g., biomedical implants, conductive material, catalysts, sensors, etc.) driven by surface charge under *in situ* and *in operando* conditions.

## Graphical abstract

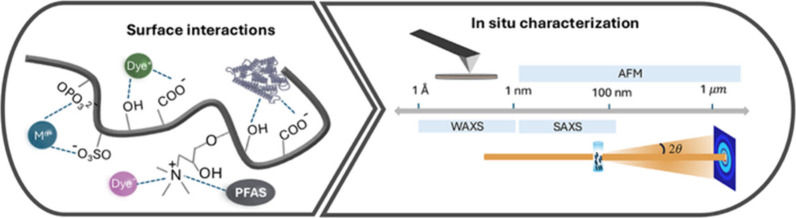

## 1 Introduction

Cellulose is a polysaccharide composed of d-anhydroglucose ring units linked by β(1→4) glycosidic bonds and has a hierarchical structure. The uniformity of the monomers and the intra- and intermolecular bonds of cellulose allow the arrangement of cellulose molecules into highly ordered elementary fibrils. These fibrils consist of alternately ordered and disordered regions along the fibril axis that align and bind to form cellulose microfibrils. The width of the elementary fibril varies depending on the source and is in the range of a few nanometers.^1–3^ Nanocellulose is typically isolated from cellulosic biomass (plants, bacteria, tunicate, bioresidues, and textile waste) by a top-down approach and gives rise to two types of nanomaterials, viz., cellulose nanocrystals (CNCs) and cellulose nanofibers (CNFs).^1,2^ CNFs and CNCs refer to the ISO 20477:2023 established terms and definitions for nanocellulose.^4^ Typically, mechanical processes on cellulose in an aqueous medium produce CNFs, made up of individual fibrils or bundles of elementary fibrils, and an acid hydrolysis process to remove the disordered regions in the cellulose chains gives rise to CNCs. Enzymatic hydrolysis has also been employed in some cases to reach nanoscale cellulose materials. On the other hand, bacterial cellulose (BC) is biosynthesized nanocellulose produced through a bottom-up approach and has high purity and crystallinity. We point to our recent article for further details on nanocellulose classifications.^5^

The atomic force microscopy (AFM) images in Figure 1a–d reveal the typical morphologies of cellulose nanofibers and cellulose nanocrystals from wood; CNFs include ordered and disordered regions, whereas CNCs have the disordered regions removed by chemical processes such as acid hydrolysis. During nanocellulose processing by chemical and mechanical treatments,^6,7^ its surface chemistry can be specifically modified to add distinct functionalities.^8^ Water treatment is an emerging application for nanocellulose, driven by its high surface area, versatile surface chemistry, and nanostructured morphology. Anionic surface groups (−HSO_3_^–^, −OPO_3_^2–^, or COO^–^) can interact and adsorb positively charged entities such as metal ions, dyes, etc.^9^ When nanocellulose is functionalized using negative functional groups, the adsorption of anionic species such as nitrates, sulfates, and anionic dyes can occur. Over the past decade, extensive research has demonstrated the potential of nanocellulose as a building block to develop porous membranes,^10,11^ owing to the ease of pore-size tunability, as well as the reactive surface that allows the introduction of anionic and cationic groups for pollutant capture.^10^ In addition, it has also been shown to display antifouling activity that is attributed largely to its hydrophilic nature and surface chemistry.^12–14^ Figure 1e shows the growing trend in increased publications in this field since 2010.

**Figure 1:**
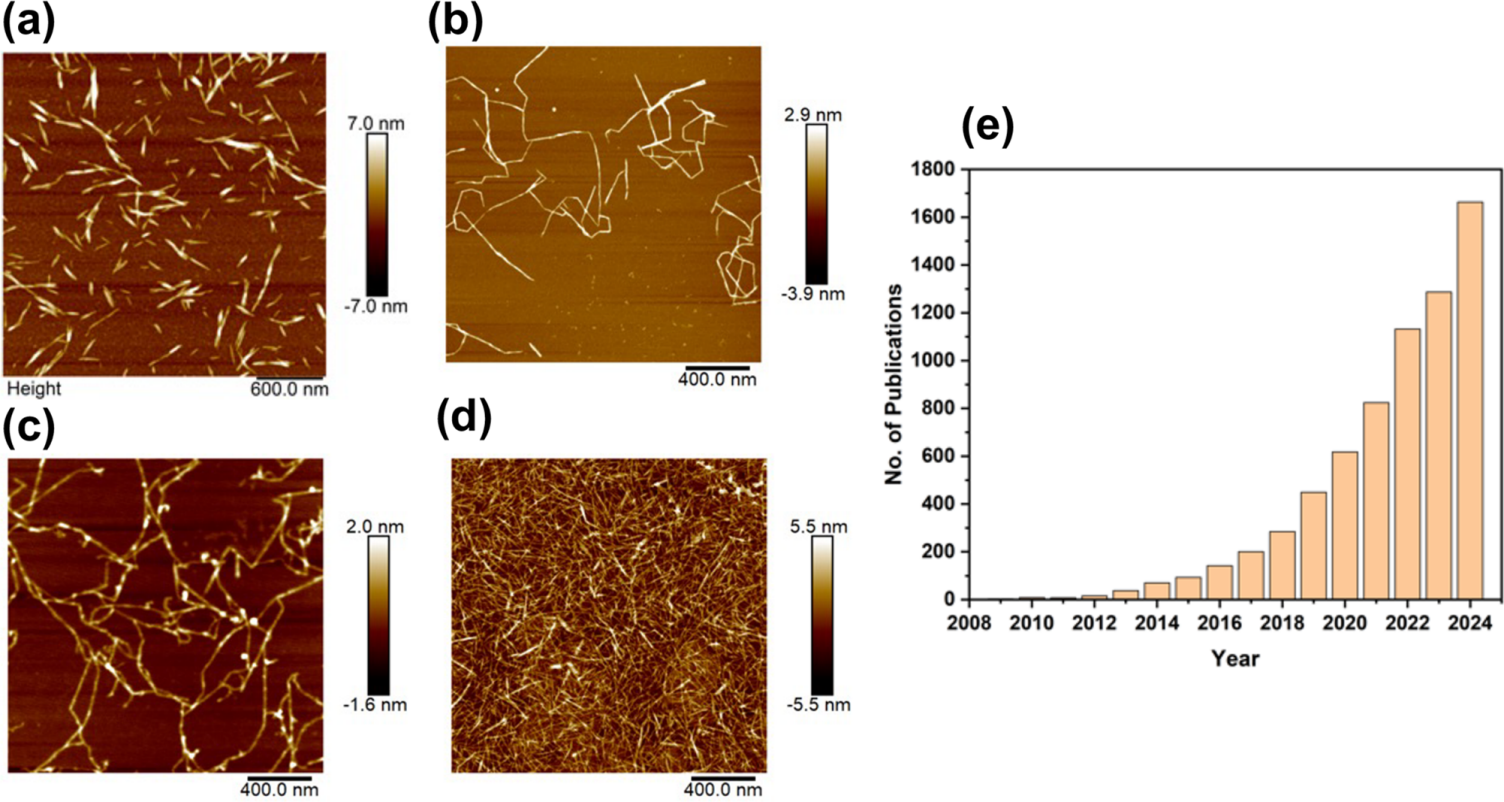
AFM images of (a) anionic CNC with carboxyl groups, (b) anionic CNF with carboxyl groups, (c) anionic CNF with phosphoryl groups (d) cationic CNF with quaternary ammonium groups respectively and (e) metrics showing the growing trend in publications covering nanocellulose use in water treatment, source (scopus, key words: nanocellulose + water treatment).

Recent research has also shown that nanocellulose with carboxyl groups, lignin residues, or zwitterionic grafts exhibits antifouling as well as antibacterial properties.^15^ Antibacterial properties may be related to the presence of functional groups toxic to bacteria, its influence in cell division and bacterial cell walls, or other complex biological phenomena in the presence of nanoparticles (NPs). This is found to differ across different nanocellulose species but is not understood sufficiently.

Moreover, CNCs or CNFs act as a universal template for hybrid synthesis and, in combination with species such as metal organic frameworks^10^ (MOFs), graphene oxide (GO),^16^ TiO_2_ nanowires,^17^ etc., offer efficient water treatment through charge-specific adsorption, size exclusion, antifouling, antibacterial properties, photocatalytic properties, magnetic properties, and combinations thereof. These hybrids combine the charge-specific adsorption of nanocellulose with enhanced moisture stability, decreased swelling, enhanced water flux, photocatalytic activity, magnetic properties, selectivity toward target pollutants, antifouling, antibacterial properties, as well as regeneration capability, all needed for enhanced membrane performance. How the chemical and structural transformations during hybrid synthesis affect the resultant functionalities (synergistically or adversely) is understood only to a limited extent to date. Charge-specific attraction or repulsion is considered the main mechanism of pollutant removal while using nanocellulose based adsorbents. However, nanocellulose hybrids present multiple pathways for the removal of water pollutants, and the related mechanisms, competitions, and synergies at nano- and subnanoscales are not sufficiently explored. Furthermore, the mechanisms involved when real water samples containing multiple pollutant types are present have not been evaluated to date.

Unraveling many of the above interactions/reactions that occur in a moist environment is challenging, and the characterization techniques under dry conditions provide insufficient (or even incorrect) insight into the chemical and physical mechanisms involved. Therefore, *in situ* and *in operando* characterization methods for studies in aqueous environments are essential to be developed and validated.

*In situ* SAXS/WAXS provides an opportunity to collect information on structural and chemical interactions, whereas AFM gives possibilities for imaging in the dry and liquid phase as well as mapping mechanical properties and molecular level-specific interactions (adhesion, repulsion, adsorption/desorption) in aqueous environments. Herein we summarize the works done in our group on two techniques, viz., (i) atomic force microscopy and (ii) synchrotron scattering studies, used for the *in situ* characterization of nanocellulose based materials in the context of water treatment. It may be noted that these two techniques can be complemented with other techniques such as advanced microscopy (OM, liquid phase HR-SEM, and liquid phase HR-TEM) to gain morphological, chemical, and crystallographic insights, whereas spectroscopy (FTIR and Raman) and *in situ* XANES (X-ray absorption near edge structure) can be used to gain a molecular level understanding and determine oxidation states; however, these are not covered in this Account.

## 2 *In Situ* AFM for Probing Nanocellulose–Contaminant Interactions

Atomic force microscopy (AFM) is a versatile technique that has been extensively applied for surface analysis with possibilities for advanced topographical imaging with micro to subnanometer scale resolution and force measurements with piconewton sensitivity, even in dry and liquid media.^12,14,18^ Its broad utility stems from its ability to characterize a range of properties, including surface morphology, topography, roughness, nanomechanical properties, and interfacial interaction force. Figure 2 shows the use of AFM applied to nanocellulose in an aqueous environment, where imaging during adsorption, surface interactions, and nanomechanics is represented. Furthermore, the unique capabilities of the advanced quantitative AFM modes^13^ and AFM based infrared spectroscopy (AFM-IR)^19^ provide chemical analysis and compositional mapping with spatial resolution far below conventional optical diffraction limits.

**Figure 2:**
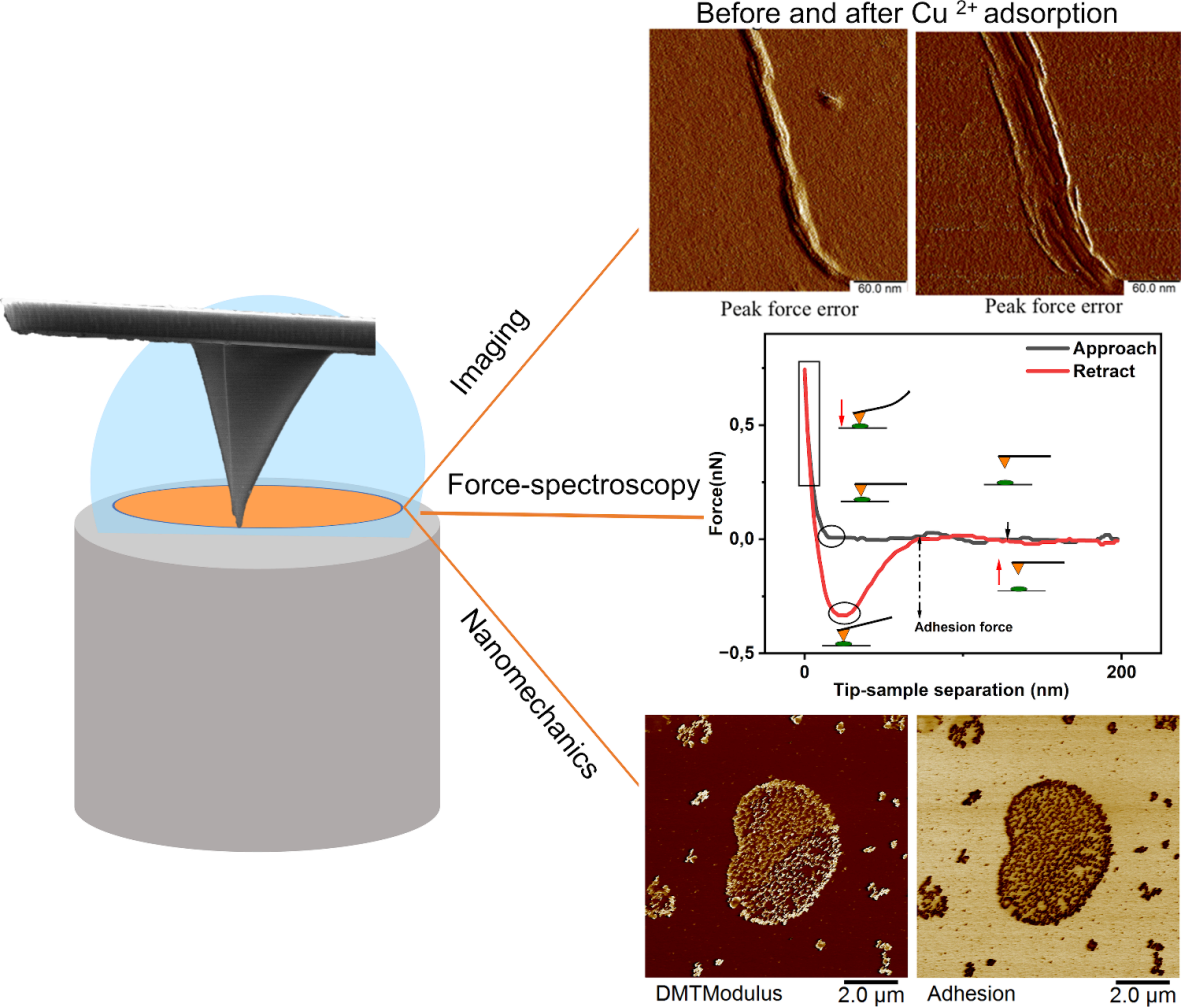
Overview of the AFM capabilities related to imaging during adsorption, surface interactions, and nanomechanics applied to nanocellulose in an aqueous environment. (The AFM images are reproduced with permission from the ref 20. Copyright 2017 Royal Society of Chemistry. The force spectroscopy image is adapted with permission from the ref 21. Copyright 2024 American Chemical Society.) The imaging panel shows cellulose nanofibers in liquid before and after Cu^2+^ adsorption. The force spectroscopy panel depicts approach–retraction cycles of a functionalized AFM probe with nanocellulose and BSA. The nanomechanics panel displays DMT modulus and adhesion maps of BSA.

### 2.1 AFM Studies on Nanocellulose Materials under Dry Conditions

AFM of nanocellulose based water treatment materials under dry conditions was studied by Mathew and co-workers. Zhu et al.^22^ studied the self-assembly of nanocellulose and graphene oxide and their copper adsorption. The AFM imaging in the dry state revealed that GO nanoparticles of small size exhibit a tendency to anchor onto and align along single TEMPO oxidized CNF (TOCNF) strands, whereas the elongated and flexible TOCNFs envelop the large, planar GO surfaces. This mutual interaction drives the self-organization of a noncrystalline, porous biohybrid architecture. In another work we used AFM to reveal information about the nanomechanical properties of the hybrid material, which shows an enhanced modulus compared to the pristine membrane.^14^ AFM has been used to evaluate the surface roughness of the membranes, which could reveal that a reduced fouling was associated with reduced surface roughness after a nanocellulose coating is applied to a commercial membrane, as well as the microscale porosity of the membrane and the uniformity of the coating.^15,23^

AFM with colloidal probes has been widely employed to investigate cellulose based systems under dry conditions. For instance, the examination of adsorption behavior on nanocellulose surfaces can reveal the formation of contaminant layers. Zhu et al. demonstrated that copper ions form clustered layers on the surface of TOCNF.^20^ Following copper ion adsorption, adhesion forces increased 3-fold, and the nanofibers became stiffer. A comparable trend was observed in hybrid materials containing TOCNF and GO. Pettersson and co-workers also used AFM to study interactions between 2D MXene and nanocellulose, where the nanocellulose was coated onto a silica sphere and brought into contact with a substrate containing the target material.^24^ A similar approach was used by the same group to study interactions involving graphene, which is particularly relevant for developing hybrid materials.^25^

### 2.2 AFM Studies on Nanocellulose in Liquid Environments

We argue that one of the major advantages of AFM is its capability to operate within liquid environments, which is highly beneficial for replicating real-world conditions found in water treatment systems, such as varying pH^26^ and ionic strengths.^27^ *In situ* liquid phase atomic microscopy based methods are developed and used by Mathew and co-workers as a powerful tool to evaluate nanocellulose and its hybrids in aqueous environments. The group has a leading position in the development and use of *in situ* techniques for probing nanocellulose based membranes in aqueous media. Imaging nanocellulose under liquid conditions allows for the observation of dynamic structural changes, including fibril swelling and reorganization, as demonstrated in our earlier work.^20^ Force spectroscopy using AFM enables the quantitative analysis of mechanical properties, such as stiffness and Young’s modulus, as well as interfacial phenomena including adhesion, repulsion, and binding energies between surfaces.^28,29^ Our research has successfully applied these capabilities to study interactions between nanocellulose and various substances in a liquid environment. AFM colloidal probes, typically comprising spherical particles ranging in size from 1 to 50 μm, are affixed to the end of microfabricated cantilevers. These probes offer several advantages in force spectroscopy experiments. For instance, their well-defined, symmetrical geometry allows for a more precise quantification of interaction forces and enables normalization of measurements across different experimental conditions, facilitating comparisons and the application of theoretical models.^30^ However, one trade-off is their relatively large contact area with the sample, which results in a lower spatial resolution compared with sharp AFM tips. We have successfully functionalized AFM tips with TOCNF or TOCNC and used them in understanding the interactions with water pollutants. We directly measured the interaction forces with Victoria Blue dye immobilized on a mica surface. Additionally, we utilized an indirect method to evaluate the binding behavior between functionalized nanocellulose surfaces and copper ions. These experiments underscore the strength of AFM as a dual-purpose technique, offering both high-resolution imaging and precise force measurements for exploring nanocellulose behavior in complex environments.

Other commendable works where nanocellulose was probed using AFM in aqueous environments come from the group of Österberg. Experiments were conducted in 0.1 and 10 mM NaCl solutions at pH 6.5, where they focused on the antibacterial properties of nanocellulose surfaces, revealing that a force of 50 nN was sufficient to disrupt bacterial cell walls.^31^ Nugroho et al.^32^ used colloidal probe AFM to analyze the adhesion between various biomimetic films: collagen I, collagen IV, laminin 521, and cellulose nanofibrils in PBS. They observed the strongest adhesion between collagen IV and laminin 521, with adhesion increasing over time. While cellulose nanofibrils showed weak self-adhesion, they exhibited moderate interaction with collagen. These findings contribute to the understanding of biomaterial interactions, aiding the design of scaffolds for 3D cell culture and tissue engineering. The importance of the interfacial interaction in biobased nanomaterials was the subject of a review by Österberg et al.,^33^ where they discussed in-depth analysis of how interfacial interactions influence the properties and applications of biomass-derived nanomaterials, particularly nanocellulose and lignin nanoparticles.

### 2.3 AFM Studies in Liquid Environments: Case Example of Antifouling

Beyond the general studies described above under liquid conditions, several groups have specifically focused on antifouling applications. These are summarized below as a representative example of the capability of AFM in probing pollutant interactions in aqueous media. Biofouling remains a significant and persistent issue in water treatment technologies, primarily due to the accumulation and deposition of organic matter on filtration membranes and adsorbent surfaces. This unwanted buildup leads to a gradual deterioration of membrane performance, manifesting in issues such as reduced adsorption efficiency and the blockage of adsorbent pores, which ultimately compromises the overall effectiveness of the water purification process. To better understand and replicate the mechanisms underlying this phenomenon, atomic force microscopy has been effectively utilized in experimental setups that mimic the biofouling conditions in a controlled environment. In such setups, a model foulant compound is chemically or physically attached to an AFM colloidal probe, while a nanocellulose based membrane is immobilized on a substrate within a liquid cell. This configuration allows the movement of the functionalized colloidal probe to simulate the dynamic interactions between foulants and membrane surfaces as they occur in real-world water treatment applications.^34^ In our research, we chose to use a sharp AFM tip instead of a colloidal probe. This tip was coated with cellulose nanofibers exhibiting two different surface chemistries in order to study their interactions with bovine serum albumin (BSA), a model protein often used to evaluate fouling behavior. This approach allowed us to assess the antifouling properties of different nanocellulose functionalizations with high spatial precision. In a study conducted by Eskhan et al.,^35^ two distinct types of model biofoulants were attached onto colloidal probes and used to investigate their interactions with various polymer based membrane surfaces. The adhesion forces recorded during these interactions were analyzed using extended (X)­DLVO theory, providing insights into the nature and strength of foulant membrane adhesion under different conditions.

Our group also evaluated the interaction of nanocellulose with different surface chemistries with *Escherichia coli* (*E. coli*) and its impact on antifouling. *In situ* PeakForce quantitative nanomechanical mapping (PFQNM) revealed that the surface roughness and stiffness of *E. coli* were affected when in direct contact with the nanocellulose during incubation, whereas the cells attached to CNCs promoted strong adhesion and even the embedding of *E. coli*.^36^ More recently, an innovative method for producing AFM colloidal probes was developed by Mark et al.^37^ This technique involves the use of soft hydrogel particles as probe materials, addressing many of the limitations associated with traditional rigid probes made from silica or polystyrene. Rigid probes are notoriously difficult to functionalize due to their extremely small surface area and fixed geometry. In contrast, the hydrogel based approach leverages a microfluidic aspiration system to draw soft particles into the AFM cantilever, offering greater flexibility in probe customization and enabling the use of nanoscale colloids. This advancement significantly broadens the potential applications of AFM in studying realistic fouling processes and interactions in water treatment systems, especially at scales relevant to industrial implementation. A summary of previous work is provided in Table 1.

**Table 1:** Summary of Previous Work from the Group on *In Situ* AFM of Nanocellulose in Water Treatment Applications and Complementary *Ex Situ* Techniques and Modeling Approaches

						Complementary methods		
Type of contaminants	Pollutant/foulant	Nanocellulose type	Method	Probe functionalization	Observations	*Ex situ*	Modeling	Ref
Dyes	Victoria Blue	TOCNF, CNC	Victoria Blue (direct method)	Chemical	Higher adhesion force for TOCNF compared to CNC	SEM confirmed functionalization of the tip	The dye adopted a flat orientation to maximize its adsorption	^38^
				APTES (conical probe)				
Metal ions	Ag(I)	-Native cellulose (CM)	Silica surface in AgNO_3_ solution (indirect method)	Gluing method (spherical)	-No adhesion force for CM	SEM, XPS, and FTIR confirm the presence of silver and possible clustering of Ag(I) nanoparticles	Revealed clustering for Ag(I) on PO_3_-functionalized cellulose	^26^
		-Sulfated cellulose (SCM)			-Decreased AF with pH			
		-Phosphate cellulose (PCM)			-Increased AF with pH			
	Cu(II)	TOCNF on mica coated with APTES	Cu(II) (indirect method)	Chemical method (conical)	Adhesion force increased from 50 to 140 pN after Cu(II) adsorption	AFM, SEM-EDS, and XPS indicate a copper nanolayer on TOCNF	-	^20^
	Cu(II)	TOCNF/GO nanoparticles	Cu(II) (indirect method)	Chemical method (conical)	-	AFM imaging showed self-assembly of the biohybrid	Evaluated the nature of aggregation and pore size	^14,22^
	Cu(II)	TOCNF, CNC	Cu(II) (indirect method)	Chemical method (conical)	Higher adhesion force for TOCNF compared to CNC	-	Tendency of Cu(II) to self-organize by forming nanoclusters of variable size	^38^
Proteins	BSA	PCNF, TOCNF	BSA immobilized on a mica substrate	Chemical method (conical)	Low adhesion toward BSA	Fluorescence confirms the probe functionalization	-	^21^
Bacteria	*E. coli*	TOCNF, LCNC	*E. coli* immobilized on mica	Chemical method (conical)	Increase of cell wall rigidity indicating cell distress	-Imaging and PFQNM	-	^36^
						-Biological studies		

## 3 *In Situ* SAXS/WAXS Studies on Nanocellulose Based Water Treatment Materials

### 3.1 How Synchrotrons Work and Their Significance

Synchrotron radiation (SR), which started as a nuisance due to energy loss in particle accelerators built for nuclear and particle physics experiments, has now provided researchers with endless possibilities to investigate the fundamentals of nanoscale systems under *in situ* conditions. In brief, in a synchrotron facility, electrons are first accelerated to near-light speed in a linear accelerator, followed by transmission into a storage ring, where they are steered by a series of bending and focusing magnets. As electrons pass through specially designed magnetic insertion devices such as wigglers and undulators, they undergo periodic oscillation, resulting in the emission of synchrotron radiation, which is highly collimated and tunable. This SR, covering a wide spectral range from infrared to X-rays, is then directed through beamlines to experimental end stations (Figure 3). Compared with lab based methods, synchrotron characterization approaches provide several benefits. In general, gathering data at a beamline happens far more quickly. It takes minutes or even seconds compared to hours with lab based instrumentation. Because of the extreme brightness of synchrotron radiation, the quality of the data is much improved and allows for the probing of samples at low concentrations.^39^

**Figure 3:**
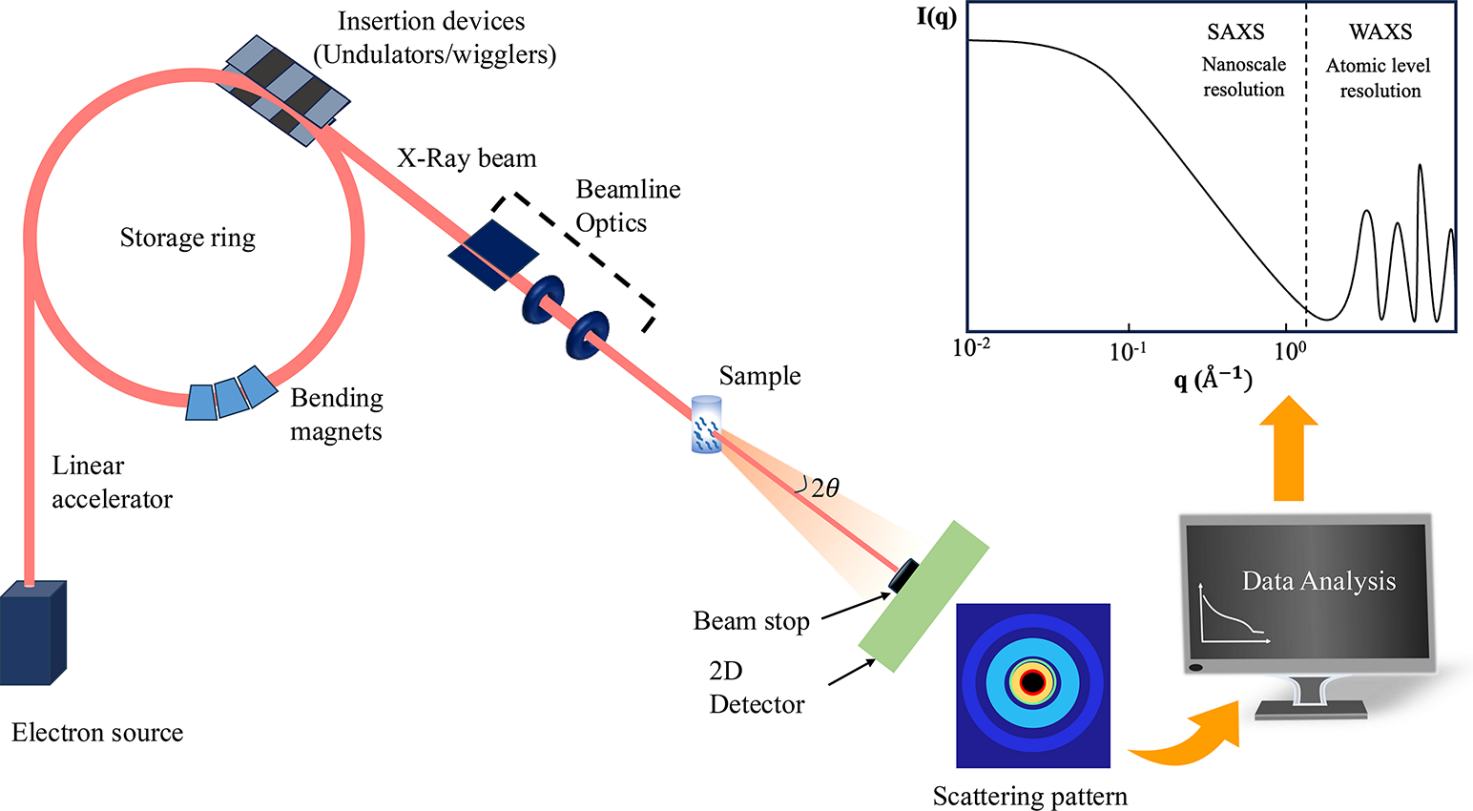
Schematic diagram of a beamline and workflow of SAXS/WAXS analysis. The resulting scattering patterns are analyzed to obtain structural information across different length scales, with SAXS probing nanoscale features and WAXS revealing atomic level structures.

Synchrotron X-ray scattering, a reciprocal space scattering technique, has been widely used to characterize detailed structures of nanocellulose materials from the atomic to the nanoscale. It can be of small-angle X-ray scattering (SAXS) and wide-angle X-ray scattering (WAXS). While SAXS provides structural details of materials at the nanoscale, WAXS provides details at the atomic scale. SAXS measures coherent scattering at small angles and at smaller *q*-values to investigate structures at meso length scales. In contrast, WAXS measures the scattering at larger angles and larger *q*-values to provide structural information at the atomic level. SAXS can analyze shapes, sizes, organized structures, surface details, and in general electron density fluctuations in dimensions from 1 nm to a few hundred nanometers.^40^ WAXS provides data regarding the crystallinity, crystallite size, and crystalline orientation of the samples.^41^ X-ray scattering experiments involve illuminating a sample with X-rays and detecting the scattered radiation (Figure 3). In SAXS, due to the small angles (2θ < 5°) involved where measurements are done very close to the primary beam, precise beam collimation, proper alignment, minimal scatter, and long sample to detector distances are crucial. Therefore, the crucial components of the instrument are the sample being contained in a cell with the least amount of scattering, multiple slits properly aligned to create a collimated beam, and an assessed beam path.^40^ A beam stop, usually made of highly X-ray-absorbing material, such as lead, protects the detector from the direct beam. Beam stops, which incorporate an X-ray sensor such as a pin diode, allow intensity calibrations based on sample absorbance.^42^ WAXS has the same instrumentation, but the sample to detector distances are much shorter.

The precise characterization of nanocellulose materials presents a significant challenge when relying on traditional microscopic techniques due to their size and small changes in surface chemistry depending on the source and extraction technique. These microscopy techniques are inappropriate for bulk measurements due to the limited analysis area and the types of sample settings that may be investigated.^43,44^ Hence, scattering techniques have gained popularity for their ability to accurately measure the structural properties of nanocellulose as they provide structural data averaged over macroscopic volume. SAXS and WAXS, along with other complementary techniques such as small-angle neutron scattering, X-ray absorption spectroscopy, tomography, electron microscopy, and AFM, provide comprehensive insights into the morphology, orientation, and chemical and structural changes of cellulose based materials derived from various sources and processed under different conditions. Additionally, the adjustable X-ray source increases the viability of *in situ*/*operando* studies. Hence, scientific and industrial communities have the chance to alter the synthesis and manufacturing processes through learning about potential flaws in the structure and the fundamental mechanisms involved in their production as well as when their products are being used in various applications, such as water purification.

Nevertheless, SAXS/WAXS on nanocellulose in the process is scarcely reported. SAXS/WAXS has been frequently used to understand the self-assembly, alignment, and flow dynamics of nanocellulose *in situ*, providing important insights to improve the process of fabricating new cellulose based nanocomposites. For more information, see the Supporting Information.

### 3.2 Material Dynamics during Water Purification

Although nanocellulose materials are widely being used in water purification, there have been only a few *in situ* studies of nanocellulose materials during water filtration using SAXS/WAXS. *In situ* X-ray scattering (SAXS) and X-ray absorption near edge structure spectroscopy (XANES) are expected to provide a deeper understanding of the chemical (interaction forces and oxidation states) and physical aspects (morphology and particle growth) of nanocellulose hybrid membranes in terms of processing and performance.

Our group has pioneered the *in situ* characterization of nanocellulose based water filtration materials. We have also successfully demonstrated the potential to use *in situ* SAXS to evaluate the pore structure, adsorption process, and metal (oxide) cluster formation during water treatment.^45,46^ The antifouling performance of nanocellulose membranes with zwitterionic grafts is also evaluated *in operando* at PETRA III, DESY, Germany.^8^ We developed nanocellulose membranes grafted with zwitterionic poly­(cysteine methacrylate) (PCysMA) and explored the mechanism involved when these membranes interact with water and BSA as a foulant.^8^ This approach involved *in situ* SAXS measurements at the MiNaXS beamline P03 at PETRA III, DESY, Germany, allowing real-time observation of changes in the pore size during filtration processes. An increased scattering intensity change in scattering pattern was observed for both unmodified and PCysMA-grafted membranes upon the addition of water to the dry membranes, owing to the swelling of cellulose fibers, affecting the pore distribution. However, upon the further addition of water, the scattering contrast decreased as more pores filled with water, while the pore size remained relatively constant, which was suggested by the unaltered scattering profile. Upon filtering a BSA solution, unmodified membranes exhibited a significant reduction in pore size, whereas modified membranes showed minimal changes, indicating that PCysMA grafting effectively suppressed BSA adsorption and enhanced antifouling performance. By offering high-resolution molecular level data during the fouling process, time-resolved *in situ* synchrotron experimental investigations will significantly improve fouling characterization capabilities.

In a separate study we also investigated the behavior of GO layered CNF membranes during the filtration of water and metal ion solutions of AgNO_3_ and Cu­(NO_3_)_2_ using a combination of *in situ* SAXS and reactive molecular dynamics (ReaxFF) simulations.^46^ SAXS data indicated that the GO layers limit the swelling and structural deformation of the CNFs during water filtration. After redrying, the membranes partially recovered their structure, indicating some irreversible structural changes. During metal ion filtration, the CNF-GO network evolved into a highly complex mass-fractal-like structure with increased correlation lengths. SAXS data also showed the formation of metal oxide nanoparticles during the drying stage with particle sizes increasing over time, highlighting intricate interactions between CNFs, GO, and metal ions. How these nanoparticles were formed without the addition of any further reducing agents was of significant interest.

We have demonstrated the ability of TOCNF to spontaneously grow metal nanoparticles during the adsorption of metal ions from aqueous solutions, under room temperature conditions, without the addition of any reducing agent. For example, the *in situ* growth of Cu_2_O nanoparticles on TOCNF membranes occurred during the filtration of Cu­(NO_3_)_2_ solution (Figure 4a).^45^ Combining *in situ* SAXS, quartz-crystal microbalance with dissipation, and UV–vis spectroscopy, we elucidated the three-step mechanism of nanoparticle formation, involving metal ion adsorption onto carboxyl groups, growth of nanoclusters upon reduction of metal atoms, and coalescence and growth of nanoparticles. SAXS during wetting and drying indicated the formation of nanoparticles in sizes of 4–6 nm with a polydispersity of 0.2–0.3. Nanoparticle growth was observed primarily during drying, with diameters ranging from 10 to 20 nm, consistent with AFM observations. This study confirmed carboxylated CNF films as effective substrates for forming metal oxide nanoparticles during metal ion adsorption. We used *in situ* studies to verify the spontaneous growth of metal oxide nanoparticles from not only copper (Cu^2+^) ion solution but also silver (Ag^+^) and iron (Fe^3+^) ion solutions, proving the universality of the phenomenon.

**Figure 4:**
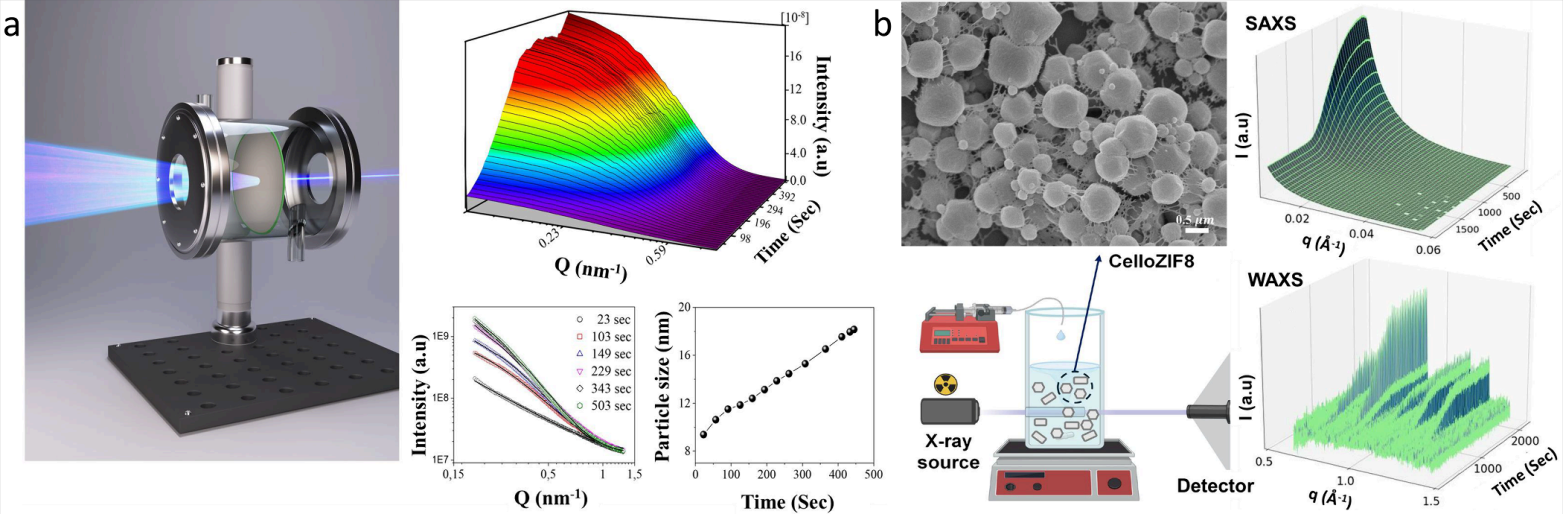
(a) Schematic representation of the experimental setup used for the SAXS measurements to demonstrate the growth of metal nanoparticles on nanocellulose membranes, the corresponding three-dimensional [*I*(*Q*) vs *Q* vs time] and two-dimensional [*I*(*Q*) vs *Q*] plots during the drying of nanocellulose films after copper adsorption, and size evolution of the Cu_2_O-NPS during drying in terms of diameter. (Reproduced with permission from ref 45. Copyright 2020 American Chemical Society.) (b) Setup used to analyze the *in situ* ZIF-8 growth on nanocellulose, SEM image of CelloZIF8, and corresponding [*I*(*Q*) vs time] SAXS and WAXS plots.

### 3.3 Mechanisms Involved in the Synthesis of Water Purification Materials

SAXS/WAXS has also been used for the *in situ*/*operando* study of the preparation of nanocellulose hybrids with the end use of water purification. TiO_2_ is an excellent photocatalyst used in water purification. Zhan et al. investigated the growth mechanism of TiO_2_ nanoparticles on a sulfated CNC scaffold using SAXS.^47^ They analyzed the reaction mixture at different time intervals and observed that the scattering peak shifts from 0.25 to 0.20 Å^–1^, implying the growth of TiO_2_ nanoparticles, and the Bragg’s peaks correspond to 2.5, 2.7, 2.9, and 3.2 nm, which were consistent with the average TiO_2_ crystal dimensions.

Cellulose-MOF systems are also a great candidate for water purification. The different pore sizes of different MOFs can be used to tune the porosities of the hybrid materials. Metilli et al. developed hybrid composites of CNCs with ZIF-8 via ultrafiltration, monitoring the time and spatial evolution of the concentration and particle orientation at the nanoscale using SAXS.^48^ The increase of scattering intensities measured close to the membrane and shift of the CNC *S*(*q*) peak toward higher *q*-values during filtration indicated the local concentration increase of CNCs and ZIF-8. Furthermore, SAXS analysis demonstrated that a higher ZIF-8 content resulted in more permeable deposits with lower flow resistance, highlighting the role of ZIF-8 in modifying the microstructure and permeability of the CNC layer. Similarly, our group has also investigated the *in situ* growth of ZIF-8 on nanocellulose using SAXS/WAXS at the MAX IV facility, and the setup used in the experiment is shown in Figure 4b.^49^

The synchrotron SAXS/WAXS techniques offer an intriguing possibility to comprehend the basics of the interfacial interactions between the filtering solution and the filter, as demonstrated by this earlier research. They can be used as tools to shed light on the mechanisms involved in the solute transport, solute accumulation in the concentration polarization layers on the filtration membranes, solute selectivity during filtration with the pore structure, and network structure changes upon filtration. Additionally, these methods can be utilized to gain a better understanding of filter fouling, which is a concern in industrial settings. A thorough understanding of how foulants interact with filter material as well as structural alterations in the foulant and filter network can result in the development of improved filters that are resistant to fouling. Overall, the significance of synchrotron SAXS/WAXS techniques lies in the fact that these characterizations can be carried out *in situ* to explore the process in real time, which is not possible otherwise; the sample preparation is straightforward; the acquisition of data is statistically averaged over a macroscopic volume; and due to higher electron density the scattering contrast for materials is much stronger.^50^ In Table 2, we summarize the *in situ*/*operando* SAXS/WAXS characterization reported for nanocellulose based water treatment materials during water filtration and material synthesis so far.

**Table 2:** Summary of *In Situ*/*Operando* SAXS/WAXS Characterization of Nanocellulose Based Water Treatment Materials

System	Contaminant	Observation	Ref
Nanocellulose membranes grafted with zwitterionic PCysMA	BSA/water	Pore structure changes upon filtering water and BSA	^8^
GO layered CNF	Cu^2+^, Ag^+^	Structure changes upon adsorption of metal ions	^46^
TOCNF	Cu^2+^, Ag^+^	Growth of nanoparticles upon metal ion adsorption	^45^
Sulfated CNC/TiO_2_	-	Growth mechanism of TiO_2_ nanoparticles	^47^
CNCs/ZIF-8	-	Time and spatial evolution of concentration and particle orientation at the nanoscale and microstructure upon deposition of CNCs/ZIF-8	^48^

## 4 Summary and Perspectives

The use of *in situ* methods under aqueous conditions is an emerging field and can have significant application not only in water treatment but also in biomedical applications. We reviewed the research publications with respect to *in situ* studies on nanocellulose based water treatment materials under aqueous conditions using *in situ* AFM and *in situ* scattering techniques.

About 12 studies are available on AFM of nanocellulose based materials in liquid environments. Only 6 of these focus on water treatment with a focus on the adsorption of water pollutants and foulants. Liquid phase AFM techniques have been successful, and structural changes, modulus changes, and interaction forces comparisons were used as evidence of the adsorption of water pollutants on nanocellulose based materials. The successful functionalization of AFM probes with nanocellulose and its use as colloidal probes are expected to open up the field further. We foresee that the next phase in this field will focus on activities to probe the site-specific nanomechanical, electrochemical, electronic, and magnetic properties in combination with imaging using bare or functionalized probes for nanocellulose based membranes in aqueous media. It is also worth mentioning that there are significant challenges related to the experimental setup for imaging in aqueous media, including modification of the AFM probe, which calls for further methodology development and improvement. From our experience, a more precise control of the nanoscopic probe, probably by using a microfluidic system and a more sophisticated micromanipulator, would be necessary to open avenues for groundbreaking experiments in this field and potentially other related fields. The key challenge here is finding the best ways to characterize and ensure that the probe coating remains stable throughout the experiment. As for the liquid cell, it needs to be improved to avoid leakage, which leads to time-consuming trial and error. Standards need to be established to determine whether the process is working properly. Improvements are also required for the calibration of deflection and spring constant measurements for functionalized probes. Finally, automation of alignment and lowering of the cost of consumables are needed to open this field to a wider user base.

While qualitative data from AFM studies can be obtained reliably to a large extent, quantitative AFM for chemical, electrochemical, and catalytic property mapping is still a significant challenge and needs development and validation. Combining AFM data with chemical mapping will be needed to further understand the mechanisms involved in adsorption at the molecular scale, and NanoIR is a potential tool in this direction.

Likewise, 352 studies are available on X-ray scattering methods of nanocellulose based materials in liquid environments with a focus on structural properties, alignment, orientation, and phase behavior. And, to the best of our knowledge, only 3 of these papers are focused on characterization during water treatment. Although SAXS/WAXS has offered endless possibilities for advanced characterizations, it is not without challenges. One must carefully control the experimental conditions to prevent beam damage that can be caused by the brilliant beam of X-rays. The energy of the X-rays and exposure time of the samples to the beam should be selected with extra carefulness. The use of attenuators to reduce beam intensity especially during beam alignment and test runs, slightly defocusing the beam to spread energy over a larger area to reduce energy density, raster scanning the sample, continuous flowing of the sample, and cryogenic cooling of the sample can help in reducing the beam damage to a great extent. Also, one should have a good understanding of the sample before performing a beamline experiment, as the X-rays can cause unfavorable reactions in the sample system, and one should consider the stability of the sample in a high energy beam. Another major challenge is data analysis of these advanced techniques. Although the rapid development of X-ray synchrotrons has led to the development of automated data analysis packages for experiments as well, these packages have limitations, such as a lack of established correction procedures that can be used to extract data and a lack of documentation of the software’s theory, making it difficult to identify steps causing discrepancies in the outputs.^50^ Hence, there is a growing demand for the development of user-friendly software for the analysis of scattering data. New software tools are being continuously developed by experts in the field.^51,52^ Advances in user-friendly data analysis methods will prevent novice users from falling into pitfalls during data processing.

In addition to the above techniques, complementary advanced microscopy (high-resolution OM, liquid phase SEM, and liquid phase TEM) as well as spectroscopy (FTIR and Raman spectroscopy) and *in situ* XANES can be used to study chemical and structural dynamics at different length scales and *in operando* conditions. Customized method development is required and has great potential to provide morphological, chemical, crystallographic, and molecular level understanding.

## Supplementary Material



## Notes

Additional literature on the use of *in situ* SAXS/WAXS on the self-assembly, alignment, and flow dynamics of nanocellulose (PDF)

## Abbreviations

AFM: Atomic force microscopy
BSA: Bovine serum albumin
CM: Native cellulose
CNCs: Cellulose nanocrystals
CNFs: Cellulose nanofibers
FTIR: Fourier transform infrared spectroscopy
GO: Graphene oxide
LCNC: Lignin containing cellulose nanocrystals
MOFs: Metal organic frameworks
NPs: Nanoparticles
PCM: Phosphate cellulose
PCysMA: Poly­(cysteine methacrylate)
PFQNM: PeakForce quantitative nanomechanical mapping
SAXS: Small-angle X-ray scattering
SCM: Sulfated cellulose
SEM: Scanning electron microscopy
SR: Synchrotron radiation
TOCNF/TOCNC: TEMPO oxidized cellulose nanofiber/crystal
WAXS: Wide-angle X-ray scattering
XANES: X-ray absorption near edge structure spectroscopy
XPS: X-ray photon spectroscopy
