# Supplementary material for: In Operando Characterization of Nanocellulose Based Water Treatment Materials Using Atomic Force Microscopy and Synchrotron Scattering
Source: Acc Mater Res. 2025 Nov 25;6(12):1451–61. doi: 10.1021/accountsmr.5c00150 (PMC12752729; doi:10.1021/accountsmr.5c00150)
Supplement: Supplementary file 1 [file mr5c00150_si_001.pdf]

Supplementary Information

In operando characterization of nanocellulose based water treatment materials using atomic force microscopy and synchrotron scattering

Houssine Khalili[a #] Senuri Kumarage[a #] and Aji P. Mathew[a, b]*

[a]Department of Chemistry, Stockholm University, Svante Arrhenius väg 16 C, Stockholm SE-10691, Sweden.

[b]Stockholm University Center for Circular and Sustainable Systems (SUCCeSS), Stockholm University, 106 91 Stockholm, Sweden.

# Both authors contributed equally

*Corresponding Author. Email: aji.mathew@su.se

S1. Additional literature on use of in situ SAXS/WAXS

Hsiao's group has explored the flow induced alignment of CNCs and CNFs using in situ scanning SAXS.[1, 2, 3] Researchers have also studied the structural organization of the CNCs ultrafiltration of a CNC suspension.[4, 5, 6] Separate studies have been carried out to analyze the combined effects of shear flow, ultrasound waves and ultrafiltration on the dynamical orientation and structural organization of cellulosic cholesteric liquid crystal suspensions using in situ SAXS.[7, 8, 9] Similarly, Bergström and group has employed time resolved SAXS to quantify the evaporation-induced nanoscale assembly of CNC in an acoustically levitating droplet.[10] In addition, co-assembly of CNC and montmorillonite (MNT) has also been carried out in a similar manner using in situ SAXS.[11] Furthermore, a systematic analysis of how the type, valency and concentration of electrolytes effect on the pitch and the chiral nematic structure of CNC has been carried by Browne et al using SAXS.[12] The influence of polymers on CNC phase behavior has also been investigated. Studies have reported the effect of both non-absorbing and adsorbing polymers on the phase behavior and self-assembly of CNCs using SAXS.[13, 14] Moreover, SAXS measurements have been employed to determine the cross-sectional dimensions of CNFs in different solvents including water, ethylene glycol, and propylene glycol suspensions.[15] Water effect on dimensions of nanocellulose crystals within bamboo fibers has been investigated using SAXS by another research group by soaking the bamboo fibers in water.[16] Similar studies of the water effect on plant cellulose fibrils have been reported in literature.[17, 18] Additionally, humidity effect on the foams of cellulose nanofibrils from upcycled cotton and wood has also been studied by in-situ WAXS and SAXS.[19] The ability of nanocellulose foams to act as superabsorbent has prompted the studies of absorption kinetic of nanocellulose foams with water and with different ionic strengths of the fluid as well.[20, 21] And studies have been carried out to elucidate the structural changes of cellulose from different origins when they are dissolved in NaOH aqueous solution.[22]

References

1. Rosén, T.; Wang, R.; He, H.; Zhan, C.; Chodankar, S.; Hsiao, B. S. Understanding ion-induced assembly of cellulose nanofibrillar gels through shear-free mixing and: In situ scanning-SAXS. *Nanoscale Adv.* **2021,** *3* (17), 4940-4951. DOI:10.1039/D1NA00236H.

2. Rosén, T.; Wang, R.; He, H. R.; Zhan, C.; Chodankar, S.; Hsiao, B. S. Shear-free mixing to achieve accurate temporospatial nanoscale kinetics through scanning-SAXS: ion-induced phase transition of dispersed cellulose nanocrystals. *Lab Chip* **2021,** *21* (6), 1084-1095. DOI:10.1039/D0LC01048K.

3. Rosén, T.; Wang, R.; Zhan, C.; He, H.; Chodankar, S.; Hsiao, B. S. Cellulose nanofibrils and nanocrystals in confined flow: Single-particle dynamics to collective alignment revealed through scanning small-angle X-ray scattering and numerical simulations. *Phys. Rev. E.* **2020,** *101* (3), 032610. DOI:10.1103/PhysRevE.101.032610.

4. Mandin, S.; Metilli, L.; Karrouch, M.; Lancelon-Pin, C.; Putaux, J. L.; Chèvremont, W.; Paineau, E.; Hengl, N.; Jean, B.; Pignon, F. Chiral nematic nanocomposites with pitch gradient elaborated by filtration and ultraviolet curing of cellulose nanocrystal suspensions. *Carbohydr. Polym.* **2024,** *337* (40), 122162-122162. DOI:10.1016/j.carbpol.2024.122162.

5. Semeraro, E. F.; Hengl, N.; Karrouch, M.; Michot, L. J.; Paineau, E.; Jean, B.; Putaux, J. L.; Lancelon-Pin, C.; Sharpnack, L.; Pignon, F. Layered organization of anisometric cellulose nanocrystals and beidellite clay particles accumulated near the membrane surface during cross-flow ultrafiltration: In situ SAXS and ex situ SEM/WAXD characterization. *Colloids Surf. A: Physicochem. Eng. Asp.* **2020,** *584*, 124030. DOI:10.1016/j.colsurfa.2019.124030.

6. Mandin, S.; Metilli, L.; Karrouch, M.; Blésès, D.; Lancelon-Pin, C.; Sailler, P.; Chèvremont, W.; Paineau, E.; Putaux, J.-L.; Hengl, N.; Bruno, J.; Frédéric, P. Multiscale study of the chiral self-assembly of cellulose nanocrystals during the frontal ultrafiltration process. *Nanoscale* **2024,** *16* (40), 19100-19115. DOI:10.1039/D4NR02840F.

7. Bosson, F.; Chèvremont, W.; Karrouch, M.; Blésès, D.; Delplace, V.; Hengl, N.; Pignon, F. In situ multiscale characterization of cellulose nanocrystals orthotropic organization achieved by combining ultrasound and frontal ultrafiltration. *Carbohydr. Polym.* **2025,** *362*, 123680-123680. DOI:10.1016/j.carbpol.2025.123680.

8. Pignon, F.; Guilbert, E.; Mandin, S.; Hengl, N.; Karrouch, M.; Jean, B.; Putaux, J. L.; Gibaud, T.; Manneville, S.; Narayanan, T. Orthotropic organization of a cellulose nanocrystal suspension realized via the combined action of frontal ultrafiltration and ultrasound as revealed by in situ SAXS. *J. Colloid Interface Sci.* **2024,** *659*, 914-925. DOI:10.1016/j.jcis.2023.12.164.

9. Pignon, F.; Semeraro, E. F.; Chèvremont, W.; Bodiguel, H.; Hengl, N.; Karrouch, M.; Sztucki, M. Orientation of Cellulose Nanocrystals Controlled in Perpendicular Directions by Combined Shear Flow and Ultrasound Waves Studied by Small-Angle X-ray Scattering. *J. Phys. Chem. C.* **2021,** *125* (33), 18409-18419. DOI:10.1021/acs.jpcc.1c03506.

10. Liu, Y.; Agthe, M.; Salajková, M.; Gordeyeva, K.; Guccini, V.; Fall, A.; Salazar-Alvarez, G.; Schütz, C.; Bergström, L. Assembly of cellulose nanocrystals in a levitating drop probed by time-resolved small angle X-ray scattering. *Nanoscale* **2018,** *10* (38), 18113-18118. DOI:10.1039/C8NR05598J.

11. Munier, P.; Di, A.; Hadi, S. E.; Kapuscinski, M.; Segad, M.; Bergström, L. Assembly of cellulose nanocrystals and clay nanoplatelets studied by time-resolved X-ray scattering. *Soft Matter* **2021,** *17* (23), 5747-5755. DOI:10.1039/D1SM00251A.

12. Browne, C.; Raghuwanshi, V. S.; Garnier, G.; Batchelor, W. Modulating the chiral nematic structure of cellulose nanocrystal suspensions with electrolytes. *J. Colloid Interface Sci.* **2023,** *650*, 1064-1072. DOI:10.1016/j.jcis.2023.07.073.

13. Sun, Q.; Lutz-Bueno, V.; Zhou, J.; Yuan, Y.; Fischer, P. Polymer induced liquid crystal phase behavior of cellulose nanocrystal dispersions. *Nanoscale Adv.* **2022,** *4* (22), 4863-4870. DOI:10.1039/D2NA00303A.
14. Cohen, N.; Ochbaum, G.; Levi-Kalisman, Y.; Bitton, R.; Yerushalmi-Rozen, R. Polymer-Induced Modification of Cellulose Nanocrystal Assemblies in Aqueous Suspensions. *ACS Appl. Polym. Mater.* **2020,** *2* (2), 732-740. DOI:10.1021/acsapm.9b01048.
15. Wang, R.; Rosen, T.; Zhan, C.; Chodankar, S.; Chen, J.; Sharma, P. R.; Sharma, S. K.; Liu, T.; Hsiao, B. S. Morphology and flow behavior of cellulose nanofibers dispersed in glycols. *Macromolecules* **2019,** *52* (15), 5499-5509. DOI:10.1021/acs.macromol.9b01036.
16. Ba, Z.; Chen, G.; Luo, H.; Luo, J. In situ SAXS analysis of the water effects on the thickness evolution of nanocellulose within bamboo fiber. *Wood Sci. Technol.* **2021,** *55* (2), 351-360. DOI:10.1007/s00226-020-01260-8.
17. Paajanen, A.; Zitting, A.; Rautkari, L.; Ketoja, J. A.; Penttila, P. A. Nanoscale mechanism of moisture-induced swelling in wood microfibril bundles. *Nano Lett.* **2022,** *22* (13), 5143-5150. DOI:10.1021/acs.nanolett.2c00822.
18. Sen, D.; Das, A.; Bahadur, J.; Bhatt, H. Time-resolved SAXS investigation on structural evolution of plant fibrillar-network during dehydration. *Surf. Interfaces* **2022,** *29*, 101737. DOI:10.1016/j.surfin.2022.101737.
19. Åhl, A.; Ruiz-Caldas, M.-X.; Nocerino, E.; Conceição, A. L. C.; Nygård, K.; McDonald, S.; Viljanen, M.; Mathew, A. P.; Bergström, L. Multimodal structural humidity-response of cellulose nanofibril foams derived from wood and upcycled cotton textiles. *Carbohydr. Polym.* **2025,** *357*, 123485-123485. DOI:10.1016/j.carbpol.2025.123485.
20. Hossain, L.; Eastman, E.; De Rango, M.; Raghuwanshi, V. S.; Tanner, J.; Garnier, G. Absorption kinetics of nanocellulose foams: Effect of ionic strength and surface charge. *J. Colloid Interface Sci.* **2021,** *601* (13), 124-132. DOI:10.1016/j.jcis.2021.05.092.
21. Hossain, L.; Raghuwanshi, V. S.; Tanner, J.; Wu, C.-M.; Kleinerman, O.; Cohen, Y.; Garnier, G. Structure and swelling of cross-linked nanocellulose foams. *J. Colloid Interface Sci.* **2020,** *568*, 234-244. DOI:10.1016/j.jcis.2020.02.048.
22. Wojtasz, J.; Bengtsson, J.; Ulmefors, H.; Bernin, D.; Östlund, Å.; Yu, S. In-situ X-ray analysis of cold alkali dissolution of cellulose pulps of various origin. *Cellulose* **2025,** *32* (1), 115-131. DOI:10.1007/s10570-024-06235-7.
